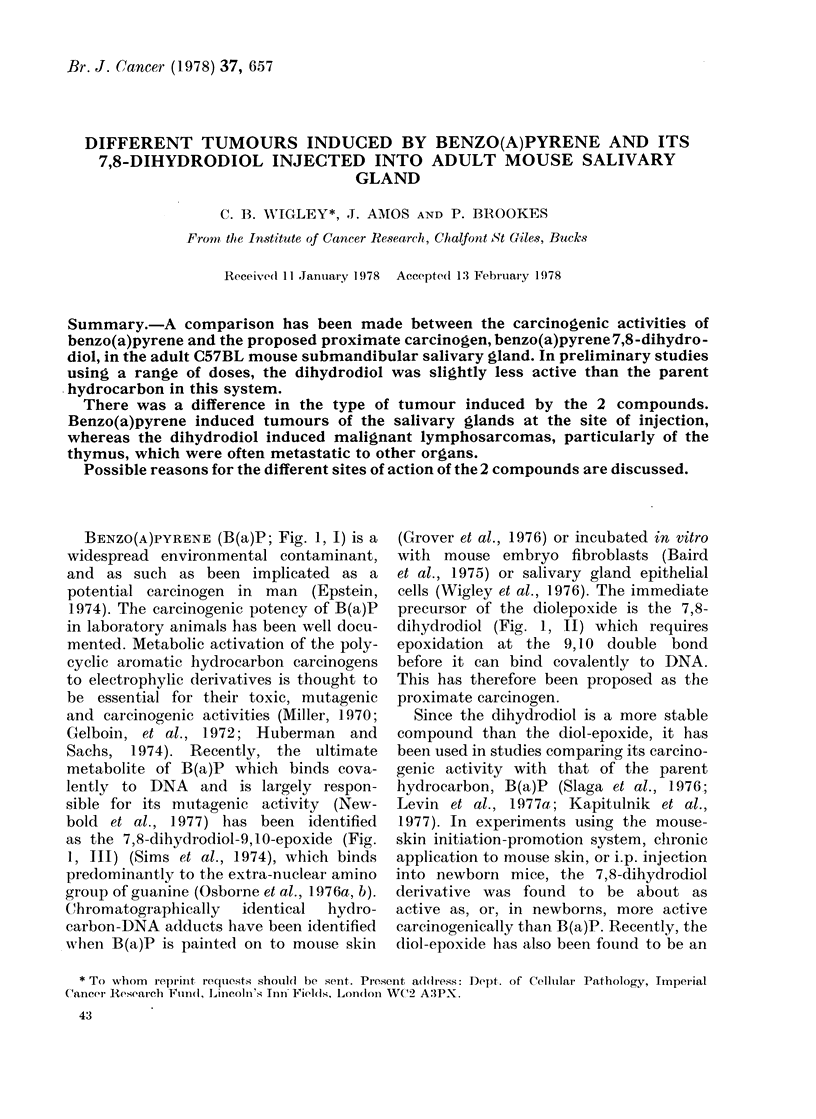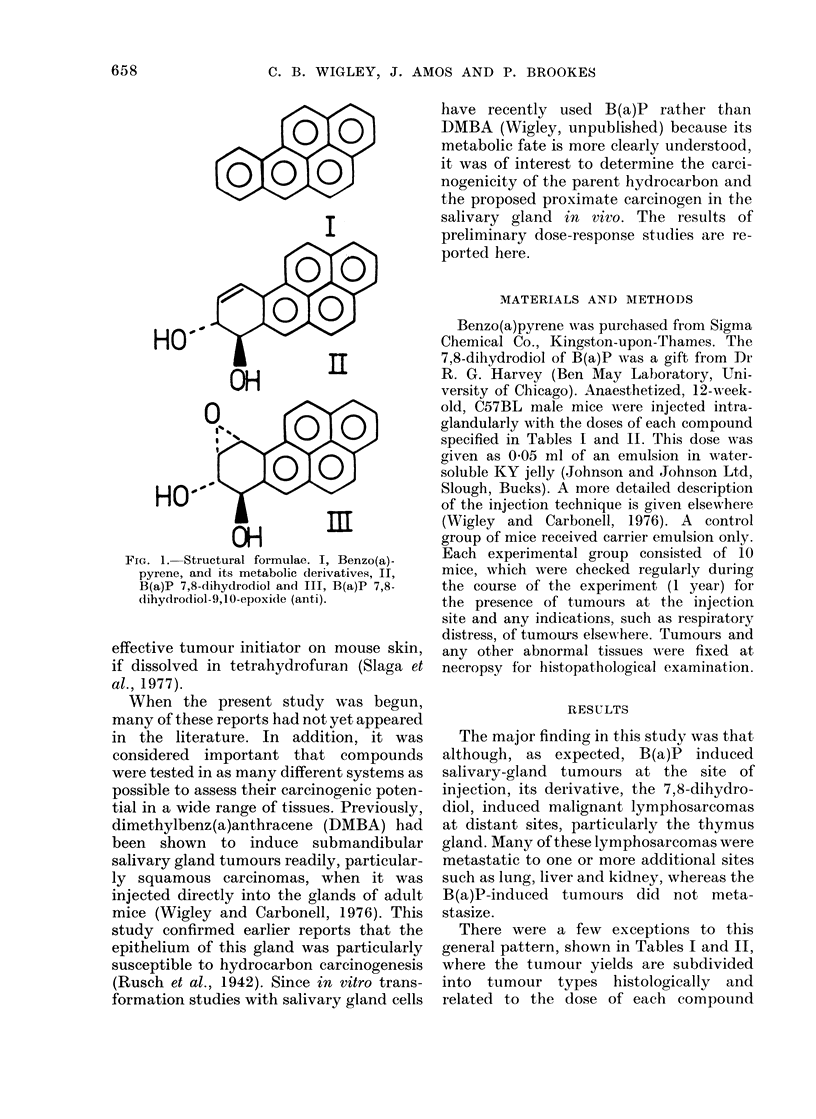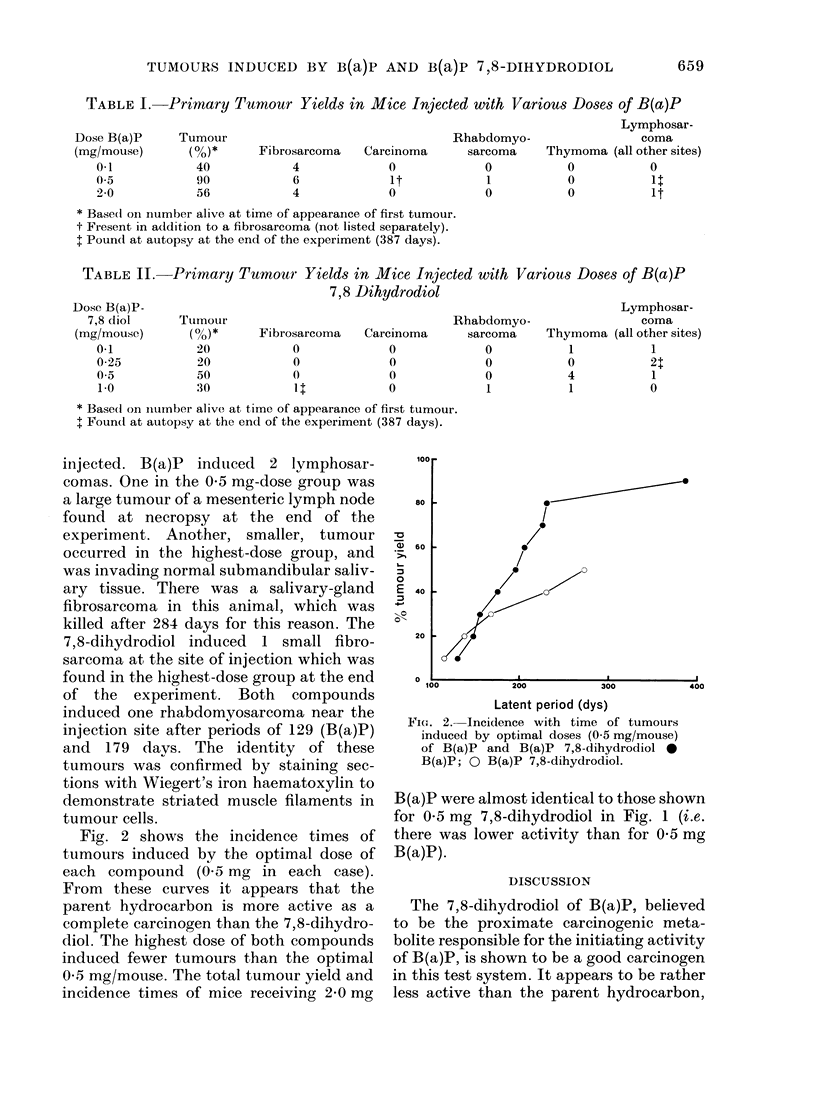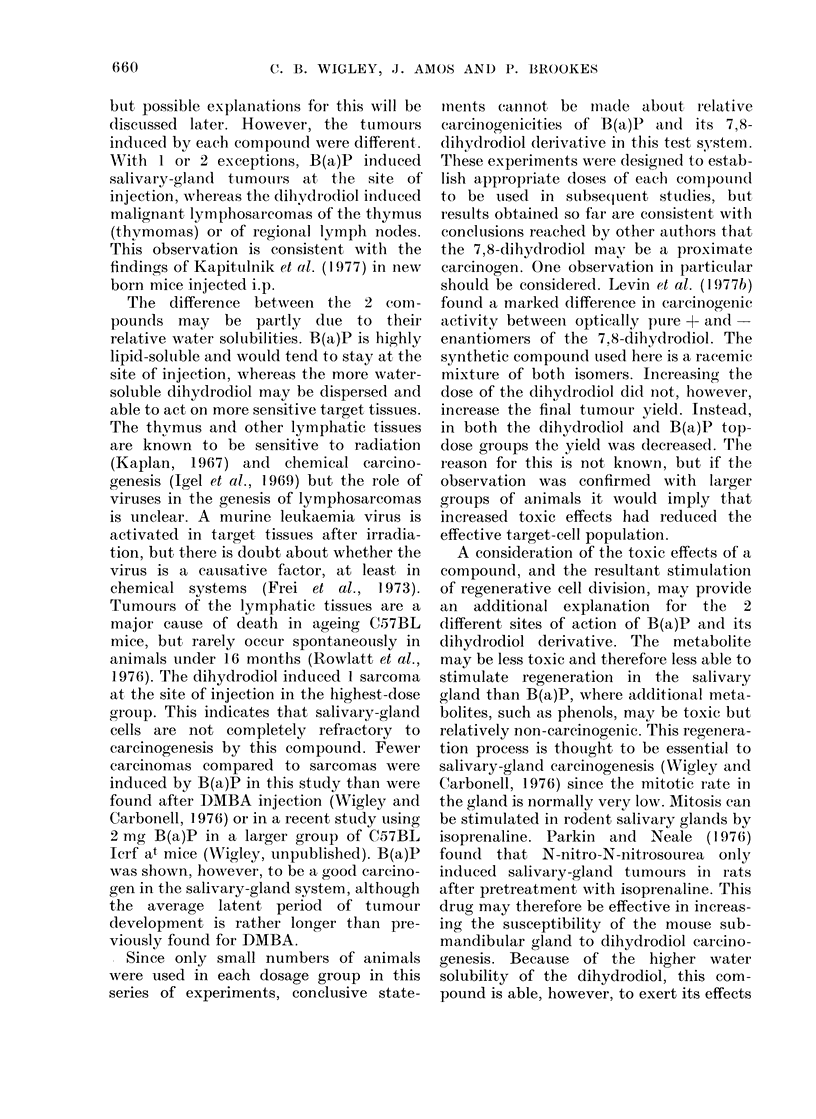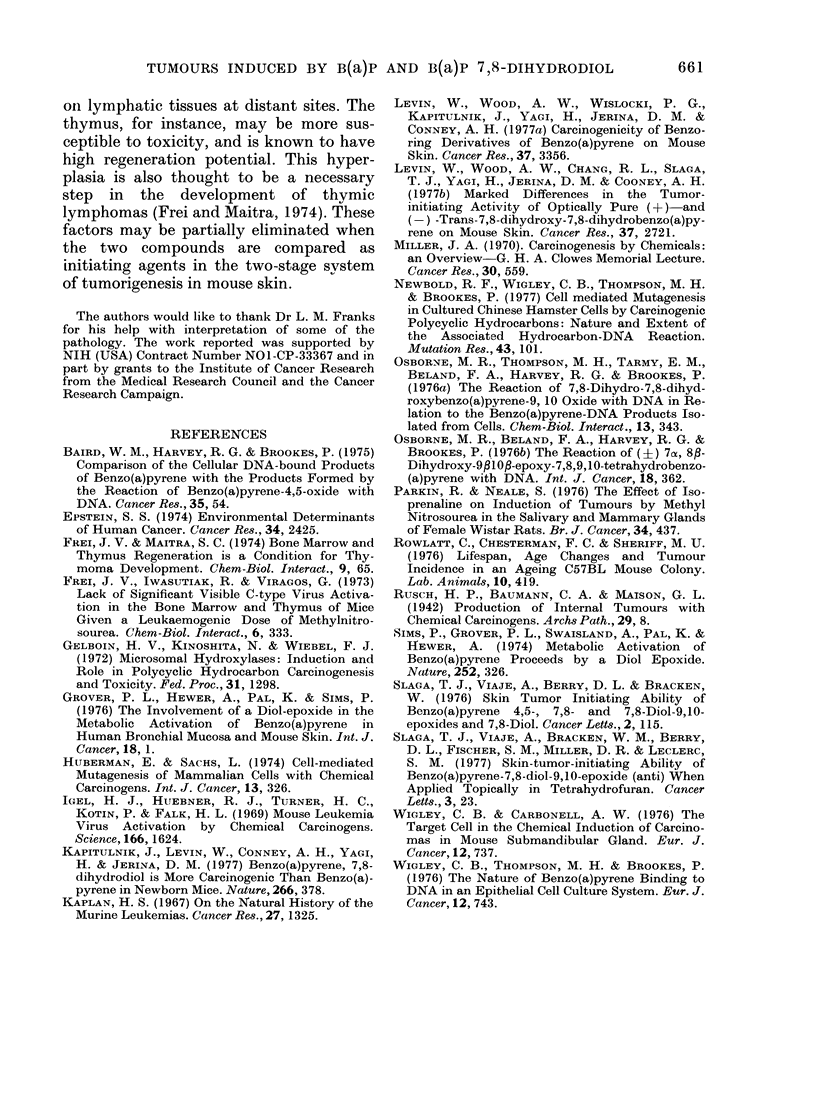# Different tumours induced by benzo(a)pyrene and its 7,8-dihydrodiol injected into adult mouse salivary gland.

**DOI:** 10.1038/bjc.1978.103

**Published:** 1978-05

**Authors:** C. B. Wigley, J. Amos, P. Brookes

## Abstract

A comparison has been made between the carcinogenic activities of benzo(a)pyrene and the proposed proximate carcinogen, benzo(a)pyrene 7,8-dihydrodiol, in the adult C57BL mouse submandibular salivary gland. In preliminary studies using a range of doses, the dihydrodiol was slightly less active than the parent hydrocarbon in this system. There was a difference in the type of tumour induced by the 2 compounds. Benzo(a)pyrene induced tumours of the salivary glands at the site of injection, whereas the dihydrodiol induced malignant lymphosarcomas, particularly of the thymus, which were often metastatic to other orgnas. Possible reasons for the different sites of action of the 2 compounds are discussed.


*Br. J. Cancer* (1978) **37**, 657

### DIFFERENT TUMOURS INDUCED BY BENZO(A)PYRENE AND ITS 7,8-DIHYDRODIOL INJECTED INTO ADULT MOUSE SALIVARY GLAND

C. B. WIGLEY*, J. AMOS AND P. BROOKES

*From the Institute of Cancer Research, Chalfont St Giles, Bucks*

Received 11 January 1978 Accepted 13 February 1978

**Summary.—A comparison has been made between the carcinogenic activities of benzo(a)pyrene and the proposed proximate carcinogen, benzo(a)pyrene 7,8-dihydrodiol, in the adult C57BL mouse submandibular salivary gland. In preliminary studies using a range of doses, the dihydrodiol was slightly less active than the parent hydrocarbon in this system.**

**There was a difference in the type of tumour induced by the 2 compounds. Benzo(a)pyrene induced tumours of the salivary glands at the site of injection, whereas the dihydrodiol induced malignant lymphosarcomas, particularly of the thymus, which were often metastatic to other organs.**

**Possible reasons for the different sites of action of the 2 compounds are discussed.**

BENZO(A)PYRENE (B(a)P; Fig. 1, I) is a widespread environmental contaminant, and as such as been implicated as a potential carcinogen in man (Epstein, 1974). The carcinogenic potency of B(a)P in laboratory animals has been well documented. Metabolic activation of the polycyclic aromatic hydrocarbon carcinogens to electrophylic derivatives is thought to be essential for their toxic, mutagenic and carcinogenic activities (Miller, 1970; Gelboin, *et al.*, 1972; Huberman and Sachs, 1974). Recently, the ultimate metabolite of B(a)P which binds covalently to DNA and is largely responsible for its mutagenic activity (Newbold *et al.*, 1977) has been identified as the 7,8-dihydrodiol-9,10-epoxide (Fig. 1, III) (Sims *et al.*, 1974), which binds predominantly to the extra-nuclear amino group of guanine (Osborne *et al.*, 1976*a*, *b*). Chromatographically identical hydrocarbon-DNA adducts have been identified when B(a)P is painted on to mouse skin (Grover *et al.*, 1976) or incubated *in vitro* with mouse embryo fibroblasts (Baird *et al.*, 1975) or salivary gland epithelial cells (Wigley *et al.*, 1976). The immediate precursor of the diolepoxide is the 7,8-dihydrodiol (Fig. 1, II) which requires epoxidation at the 9,10 double bond before it can bind covalently to DNA. This has therefore been proposed as the proximate carcinogen.

Since the dihydrodiol is a more stable compound than the diol-epoxide, it has been used in studies comparing its carcinogenic activity with that of the parent hydrocarbon, B(a)P (Slaga *et al.*, 1976; Levin *et al.*, 1977*a*; Kapitulnik *et al.*, 1977). In experiments using the mouse-skin initiation-promotion system, chronic application to mouse skin, or i.p. injection into newborn mice, the 7,8-dihydrodiol derivative was found to be about as active as, or, in newborns, more active carcinogenically than B(a)P. Recently, the diol-epoxide has also been found to be an

* To whom reprint requests should be sent. Present address: Dept. of Cellular Pathology, Imperial Cancer Research Fund, Lincoln's Inn Fields, London WC2 A3PX.

FIG. 1.—Structural formulae. I, Benzo(a)-pyrene, and its metabolic derivatives, II, B(a)P 7,8-dihydrodiol and III, B(a)P 7,8-dihydrodiol-9,10-epoxide (anti).

effective tumour initiator on mouse skin, if dissolved in tetrahydrofuran (Slaga *et al.*, 1977).

When the present study was begun, many of these reports had not yet appeared in the literature. In addition, it was considered important that compounds were tested in as many different systems as possible to assess their carcinogenic potential in a wide range of tissues. Previously, dimethylbenz(a)anthracene (DMBA) had been shown to induce submandibular salivary gland tumours readily, particularly squamous carcinomas, when it was injected directly into the glands of adult mice (Wigley and Carbonell, 1976). This study confirmed earlier reports that the epithelium of this gland was particularly susceptible to hydrocarbon carcinogenesis (Rusch *et al.*, 1942). Since *in vitro* transformation studies with salivary gland cells have recently used B(a)P rather than DMBA (Wigley, unpublished) because its metabolic fate is more clearly understood, it was of interest to determine the carcinogenicity of the parent hydrocarbon and the proposed proximate carcinogen in the salivary gland *in vivo*. The results of preliminary dose-response studies are reported here.

### MATERIALS AND METHODS

Benzo(a)pyrene was purchased from Sigma Chemical Co., Kingston-upon-Thames. The 7,8-dihydrodiol of B(a)P was a gift from Dr R. G. Harvey (Ben May Laboratory, University of Chicago). Anaesthetized, 12-week-old, C57BL male mice were injected intraglandularly with the doses of each compound specified in Tables I and II. This dose was given as 0·05 ml of an emulsion in water-soluble KY jelly (Johnson and Johnson Ltd, Slough, Bucks). A more detailed description of the injection technique is given elsewhere (Wigley and Carbonell, 1976). A control group of mice received carrier emulsion only. Each experimental group consisted of 10 mice, which were checked regularly during the course of the experiment (1 year) for the presence of tumours at the injection site and any indications, such as respiratory distress, of tumours elsewhere. Tumours and any other abnormal tissues were fixed at necropsy for histopathological examination.

### RESULTS

The major finding in this study was that although, as expected, B(a)P induced salivary-gland tumours at the site of injection, its derivative, the 7,8-dihydrodiol, induced malignant lymphosarcomas at distant sites, particularly the thymus gland. Many of these lymphosarcomas were metastatic to one or more additional sites such as lung, liver and kidney, whereas the B(a)P-induced tumours did not metastasize.

There were a few exceptions to this general pattern, shown in Tables I and II, where the tumour yields are subdivided into tumour types histologically and related to the dose of each compound

TABLE I.—*Primary Tumour Yields in Mice Injected with Various Doses of B(a)P*

| Dose B(a)P (mg/mouse) | Tumour (%)* | Fibrosarcoma | Carcinoma | Rhabdomyo-sarcoma | Thymoma | Lymphosar-coma (all other sites) |
|---|---|---|---|---|---|---|
| 0·1 | 40 | 4 | 0 | 0 | 0 | 0 |
| 0·5 | 90 | 6 | 1† | 1 | 0 | 1‡ |
| 2·0 | 56 | 4 | 0 | 0 | 0 | 1† |

* Based on number alive at time of appearance of first tumour.
† Present in addition to a fibrosarcoma (not listed separately).
‡ Pound at autopsy at the end of the experiment (387 days).

TABLE II.—*Primary Tumour Yields in Mice Injected with Various Doses of B(a)P 7,8 Dihydrodiol*

| Dose B(a)P-7,8 diol (mg/mouse) | Tumour (%)* | Fibrosarcoma | Carcinoma | Rhabdomyo-sarcoma | Thymoma | Lymphosar-coma (all other sites) |
|---|---|---|---|---|---|---|
| 0·1 | 20 | 0 | 0 | 0 | 1 | 1 |
| 0·25 | 20 | 0 | 0 | 0 | 0 | 2‡ |
| 0·5 | 50 | 0 | 0 | 0 | 4 | 1 |
| 1·0 | 30 | 1‡ | 0 | 1 | 1 | 0 |

* Based on number alive at time of appearance of first tumour.
‡ Found at autopsy at the end of the experiment (387 days).

injected. B(a)P induced 2 lymphosarcomas. One in the 0·5 mg-dose group was a large tumour of a mesenteric lymph node found at necropsy at the end of the experiment. Another, smaller, tumour occurred in the highest-dose group, and was invading normal submandibular salivary tissue. There was a salivary-gland fibrosarcoma in this animal, which was killed after 284 days for this reason. The 7,8-dihydrodiol induced 1 small fibrosarcoma at the site of injection which was found in the highest-dose group at the end of the experiment. Both compounds induced one rhabdomyosarcoma near the injection site after periods of 129 (B(a)P) and 179 days. The identity of these tumours was confirmed by staining sections with Wiegert's iron haematoxylin to demonstrate striated muscle filaments in tumour cells.

FIG. 2.—Incidence with time of tumours induced by optimal doses (0·5 mg/mouse) of B(a)P and B(a)P 7,8-dihydrodiol ● B(a)P; ○ B(a)P 7,8-dihydrodiol.

Fig. 2 shows the incidence times of tumours induced by the optimal dose of each compound (0·5 mg in each case). From these curves it appears that the parent hydrocarbon is more active as a complete carcinogen than the 7,8-dihydrodiol. The highest dose of both compounds induced fewer tumours than the optimal 0·5 mg/mouse. The total tumour yield and incidence times of mice receiving 2·0 mg B(a)P were almost identical to those shown for 0·5 mg 7,8-dihydrodiol in Fig. 1 (*i.e.* there was lower activity than for 0·5 mg B(a)P).

DISCUSSION

The 7,8-dihydrodiol of B(a)P, believed to be the proximate carcinogenic metabolite responsible for the initiating activity of B(a)P, is shown to be a good carcinogen in this test system. It appears to be rather less active than the parent hydrocarbon, but possible explanations for this will be discussed later. However, the tumours induced by each compound were different. With 1 or 2 exceptions, B(a)P induced salivary-gland tumours at the site of injection, whereas the dihydrodiol induced malignant lymphosarcomas of the thymus (thymomas) or of regional lymph nodes. This observation is consistent with the findings of Kapitulnik *et al.* (1977) in new born mice injected i.p.

The difference between the 2 compounds may be partly due to their relative water solubilities. B(a)P is highly lipid-soluble and would tend to stay at the site of injection, whereas the more water-soluble dihydrodiol may be dispersed and able to act on more sensitive target tissues. The thymus and other lymphatic tissues are known to be sensitive to radiation (Kaplan, 1967) and chemical carcinogenesis (Igel *et al.*, 1969) but the role of viruses in the genesis of lymphosarcomas is unclear. A murine leukaemia virus is activated in target tissues after irradiation, but there is doubt about whether the virus is a causative factor, at least in chemical systems (Frei *et al.*, 1973). Tumours of the lymphatic tissues are a major cause of death in ageing C57BL mice, but rarely occur spontaneously in animals under 16 months (Rowlatt *et al.*, 1976). The dihydrodiol induced 1 sarcoma at the site of injection in the highest-dose group. This indicates that salivary-gland cells are not completely refractory to carcinogenesis by this compound. Fewer carcinomas compared to sarcomas were induced by B(a)P in this study than were found after DMBA injection (Wigley and Carbonell, 1976) or in a recent study using 2 mg B(a)P in a larger group of C57BL Icrf $a^t$ mice (Wigley, unpublished). B(a)P was shown, however, to be a good carcinogen in the salivary-gland system, although the average latent period of tumour development is rather longer than previously found for DMBA.

Since only small numbers of animals were used in each dosage group in this series of experiments, conclusive statements cannot be made about relative carcinogenicities of B(a)P and its 7,8-dihydrodiol derivative in this test system. These experiments were designed to establish appropriate doses of each compound to be used in subsequent studies, but results obtained so far are consistent with conclusions reached by other authors that the 7,8-dihydrodiol may be a proximate carcinogen. One observation in particular should be considered. Levin *et al.* (1977*b*) found a marked difference in carcinogenic activity between optically pure + and − enantiomers of the 7,8-dihydrodiol. The synthetic compound used here is a racemic mixture of both isomers. Increasing the dose of the dihydrodiol did not, however, increase the final tumour yield. Instead, in both the dihydrodiol and B(a)P top-dose groups the yield was decreased. The reason for this is not known, but if the observation was confirmed with larger groups of animals it would imply that increased toxic effects had reduced the effective target-cell population.

A consideration of the toxic effects of a compound, and the resultant stimulation of regenerative cell division, may provide an additional explanation for the 2 different sites of action of B(a)P and its dihydrodiol derivative. The metabolite may be less toxic and therefore less able to stimulate regeneration in the salivary gland than B(a)P, where additional metabolites, such as phenols, may be toxic but relatively non-carcinogenic. This regeneration process is thought to be essential to salivary-gland carcinogenesis (Wigley and Carbonell, 1976) since the mitotic rate in the gland is normally very low. Mitosis can be stimulated in rodent salivary glands by isoprenaline. Parkin and Neale (1976) found that N-nitro-N-nitrosourea only induced salivary-gland tumours in rats after pretreatment with isoprenaline. This drug may therefore be effective in increasing the susceptibility of the mouse submandibular gland to dihydrodiol carcinogenesis. Because of the higher water solubility of the dihydrodiol, this compound is able, however, to exert its effects on lymphatic tissues at distant sites. The thymus, for instance, may be more susceptible to toxicity, and is known to have high regeneration potential. This hyperplasia is also thought to be a necessary step in the development of thymic lymphomas (Frei and Maitra, 1974). These factors may be partially eliminated when the two compounds are compared as initiating agents in the two-stage system of tumorigenesis in mouse skin.

The authors would like to thank Dr L. M. Franks for his help with interpretation of some of the pathology. The work reported was supported by NIH (USA) Contract Number NO1-CP-33367 and in part by grants to the Institute of Cancer Research from the Medical Research Council and the Cancer Research Campaign.

### REFERENCES

BAIRD, W. M., HARVEY, R. G. & BROOKES, P. (1975) Comparison of the Cellular DNA-bound Products of Benzo(a)pyrene with the Products Formed by the Reaction of Benzo(a)pyrene-4,5-oxide with DNA. *Cancer Res.*, **35**, 54.

EPSTEIN, S. S. (1974) Environmental Determinants of Human Cancer. *Cancer Res.*, **34**, 2425.

FREI, J. V. & MAITRA, S. C. (1974) Bone Marrow and Thymus Regeneration is a Condition for Thymoma Development. *Chem-Biol. Interact.*, **9**, 65.

FREI, J. V., IWASUTIAK, R. & VIRAGOS, G. (1973) Lack of Significant Visible C-type Virus Activation in the Bone Marrow and Thymus of Mice Given a Leukaemogenic Dose of Methylnitrosourea. *Chem-Biol. Interact.*, **6**, 333.

GELBOIN, H. V., KINOSHITA, N. & WIEBEL, F. J. (1972) Microsomal Hydroxylases: Induction and Role in Polycyclic Hydrocarbon Carcinogenesis and Toxicity. *Fed. Proc.*, **31**, 1298.

GROVER, P. L., HEWER, A., PAL, K. & SIMS, P. (1976) The Involvement of a Diol-epoxide in the Metabolic Activation of Benzo(a)pyrene in Human Bronchial Mucosa and Mouse Skin. *Int. J. Cancer*, **18**, 1.

HUBERMAN, E. & SACHS, L. (1974) Cell-mediated Mutagenesis of Mammalian Cells with Chemical Carcinogens. *Int. J. Cancer*, **13**, 326.

IGEL, H. J., HUEBNER, R. J., TURNER, H. C., KOTIN, P. & FALK, H. L. (1969) Mouse Leukemia Virus Activation by Chemical Carcinogens. *Science*, **166**, 1624.

KAPITULNIK, J., LEVIN, W., CONNEY, A. H., YAGI, H. & JERINA, D. M. (1977) Benzo(a)pyrene, 7,8-dihydrodiol is More Carcinogenic Than Benzo(a)-pyrene in Newborn Mice. *Nature*, **266**, 378.

KAPLAN, H. S. (1967) On the Natural History of the Murine Leukemias. *Cancer Res.*, **27**, 1325.

LEVIN, W., WOOD, A. W., WISLOCKI, P. G., KAPITULNIK, J., YAGI, H., JERINA, D. M. & CONNEY, A. H. (1977*a*) Carcinogenicity of Benzo-ring Derivatives of Benzo(a)pyrene on Mouse Skin. *Cancer Res.*, **37**, 3356.

LEVIN, W., WOOD, A. W., CHANG, R. L., SLAGA, T. J., YAGI, H., JERINA, D. M. & COONEY, A. H. (1977*b*) Marked Differences in the Tumor-initiating Activity of Optically Pure (+)—and (−) -Trans-7,8-dihydroxy-7,8-dihydrobenzo(a)pyrene on Mouse Skin. *Cancer Res.*, **37**, 2721.

MILLER, J. A. (1970). Carcinogenesis by Chemicals: an Overview—G. H. A. Clowes Memorial Lecture. *Cancer Res.*, **30**, 559.

NEWBOLD, R. F., WIGLEY, C. B., THOMPSON, M. H. & BROOKES, P. (1977) Cell mediated Mutagenesis in Cultured Chinese Hamster Cells by Carcinogenic Polycyclic Hydrocarbons: Nature and Extent of the Associated Hydrocarbon-DNA Reaction. *Mutation Res.*, **43**, 101.

OSBORNE, M. R., THOMPSON, M. H., TARMY, E. M., BELAND, F. A., HARVEY, R. G. & BROOKES, P. (1976*a*) The Reaction of 7,8-Dihydro-7,8-dihydroxybenzo(a)pyrene-9, 10 Oxide with DNA in Relation to the Benzo(a)pyrene-DNA Products Isolated from Cells. *Chem-Biol. Interact.*, **13**, 343.

OSBORNE, M. R., BELAND, F. A., HARVEY, R. G. & BROOKES, P. (1976*b*) The Reaction of (±) 7α, 8β-Dihydroxy-9β10β-epoxy-7,8,9,10-tetrahydrobenzo-(a)pyrene with DNA. *Int. J. Cancer*, **18**, 362.

PARKIN, R. & NEALE, S. (1976) The Effect of Isoprenaline on Induction of Tumours by Methyl Nitrosourea in the Salivary and Mammary Glands of Female Wistar Rats. *Br. J. Cancer*, **34**, 437.

ROWLATT, C., CHESTERMAN, F. C. & SHERIFF, M. U. (1976) Lifespan, Age Changes and Tumour Incidence in an Ageing C57BL Mouse Colony. *Lab. Animals*, **10**, 419.

RUSCH, H. P., BAUMANN, C. A. & MAISON, G. L. (1942) Production of Internal Tumours with Chemical Carcinogens. *Archs Path.*, **29**, 8.

SIMS, P., GROVER, P. L., SWAISLAND, A., PAL, K. & HEWER, A. (1974) Metabolic Activation of Benzo(a)pyrene Proceeds by a Diol Epoxide. *Nature*, **252**, 326.

SLAGA, T. J., VIAJE, A., BERRY, D. L. & BRACKEN, W. (1976) Skin Tumor Initiating Ability of Benzo(a)pyrene 4,5-, 7,8- and 7,8-Diol-9,10-epoxides and 7,8-Diol. *Cancer Letts.*, **2**, 115.

SLAGA, T. J., VIAJE, A., BRACKEN, W. M., BERRY, D. L., FISCHER, S. M., MILLER, D. R. & LECLERC, S. M. (1977) Skin-tumor-initiating Ability of Benzo(a)pyrene-7,8-diol-9,10-epoxide (anti) When Applied Topically in Tetrahydrofuran. *Cancer Letts.*, **3**, 23.

WIGLEY, C. B. & CARBONELL, A. W. (1976) The Target Cell in the Chemical Induction of Carcinomas in Mouse Submandibular Gland. *Eur. J. Cancer*, **12**, 737.

WIGLEY, C. B., THOMPSON, M. H. & BROOKES, P. (1976) The Nature of Benzo(a)pyrene Binding to DNA in an Epithelial Cell Culture System. *Eur. J. Cancer*, **12**, 743.